# Triptycene-based small molecules modulate (CAG)·(CTG) repeat junctions†
†Electronic supplementary information (ESI) available. See DOI: 10.1039/c5sc01595b



**DOI:** 10.1039/c5sc01595b

**Published:** 2015-06-10

**Authors:** Stephanie A. Barros, David M. Chenoweth

**Affiliations:** a Department of Chemistry , University of Pennsylvania , 231 South 34th Street , Philadelphia , PA 19104-6323 , USA . Email: dcheno@sas.upenn.edu

## Abstract

Nucleic acid three-way junctions (3WJs) play key roles in biological processes such as nucleic acid replication in addition to being implicated as dynamic transient intermediates in trinucleotide repeat sequences. Structural modulation of specific nucleic acid junctions could allow for control of biological processes and disease states at the nucleic acid level. Trinucleotide repeat expansions are associated with several neurodegenerative diseases where dynamic slippage is thought to occur during replication, forming transient 3WJ intermediates with the complementary strand. Here, we report triptycene-based molecules that bind to a d(CAG)·(CTG) repeat using a gel shift assay, fluorescence-quenching and circular dichroism.

## Abstract 2

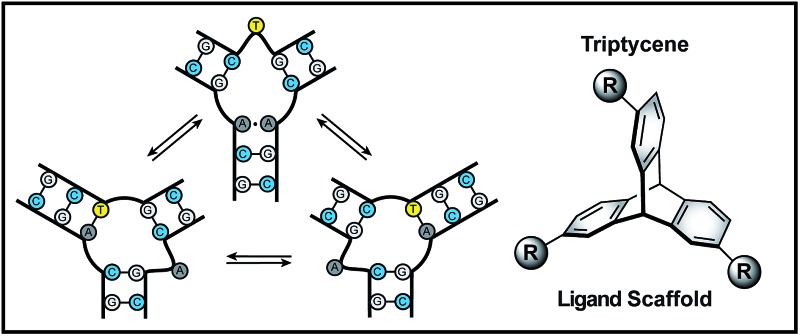
A triptycene-based scaffold is used to develop a new class of ligands for modulating the structure of junction forming trinucleotide repeat expansion sequences.

## Introduction

Nucleic acid junctions play important roles in biological processes and serve as key structural motifs in nanotechnology and aptamer-based sensing applications.^1^ In biology, three-way junctions (3WJs) are found as transient intermediates during replication, recombination, and DNA damage repair.^2^ Junctions are also present in several viral genomes, such as HIV-1, HCV, and adeno-associated virus in addition to playing key roles in viral assembly.^3^ Nucleic acid junctions are also prevalent in the emerging field of DNA and RNA nanotechnology where the bacteriophage phi29 pRNA containing RNA three-way junctions provide a particularly impressive example.^3d–g^ Furthermore, they occur in trinucleotide repeat expansions found in unstable genomic DNA associated with neurodegenerative diseases.^4^ The development of structure and sequence-specific nucleic acid binding molecules remains an important challenge in chemical biology.^5^ The ability to target specific motifs using small molecules would allow for the precise control of biological processes and the possibility of modulating disease states.

**Fig. 1:**
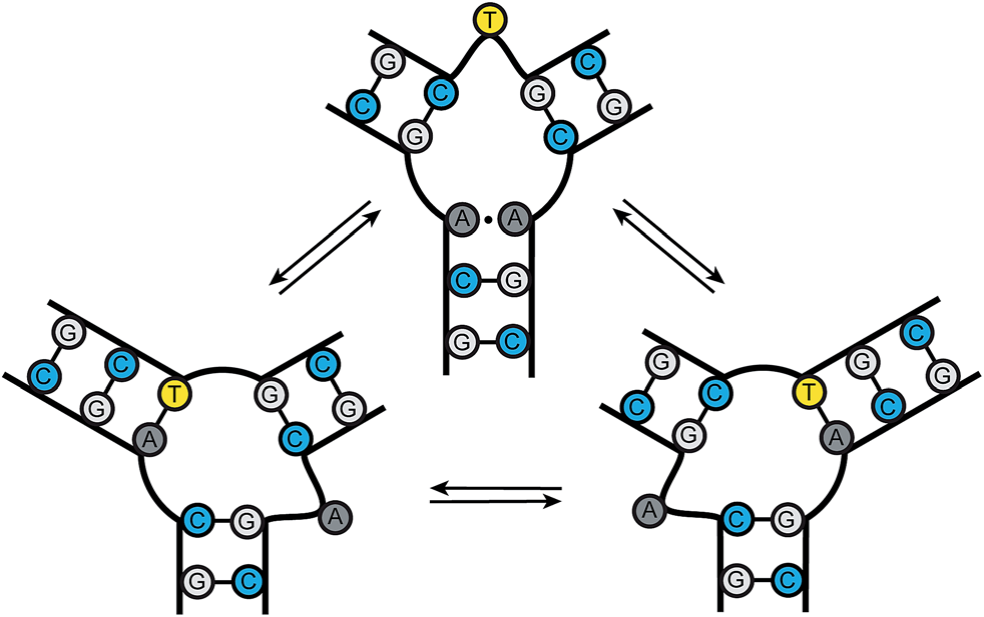
Slipped DNA junctions formed by (CAG)·(CTG) repeats.

DNA trinucleotide repeats are present throughout the genome. Expansions of these repeats, however, are associated with a number of neurodegenerative diseases, including Huntington's disease, spinobulbar muscular atrophy, and myotonic dystrophy.^6^ Current models of triplet repeat expansion disease suggest slippage during DNA synthesis by the formation of dynamic DNA hairpin structures. As the length of the repeat increases, the growing hairpin structure gains thermodynamic stability, with repeat length providing an important positively correlated diagnostic for disease severity.^6^ Slipped-out (CAG)_*n*_·(CTG)_*n*_ repeats have been implicated in the pathogenesis of triplet repeat expansion diseases such as Huntington's disease and several others.^4,6^ These “slipped-out” regions are dynamic and occur along the duplex, forming three-way junctions. Current models suggest that one arm of the junction contains the excess repeats while the other arms maximize complementary pairing between adjacent strands. A dynamic ensemble of conformations are possible at the slipped junction interface, where base pairing interactions differ with each state. The slipped-out (CAG)_*n*_ repeat in Fig. 1 has been shown to contain one unpaired base at the center of the junction. Previous NMR studies have demonstrated that this sequence can adopt a multitude of conformations, where dynamic single nucleotide bulges at the junction interface interconvert between structures.^4*c*^ Small molecule probes could provide important tools for gaining molecular level insight into the dynamics and repair processes associated with trinucleotide repeat junctions; however, probes of this kind are currently unknown.

Recently, we reported a new class of triptycene-based three-way junction (3WJ) binders.^7^ Here, we apply the triptycene scaffold as a first step toward developing new tools to recognize trinucleotide repeat junctions. We assessed the ability of our new triptycene based molecules to modulate the structure of d(CAG)_*n*_ repeats using gel shift, fluorescence quenching, and circular dichroism (CD).

## Results and discussion

**Fig. 2:**
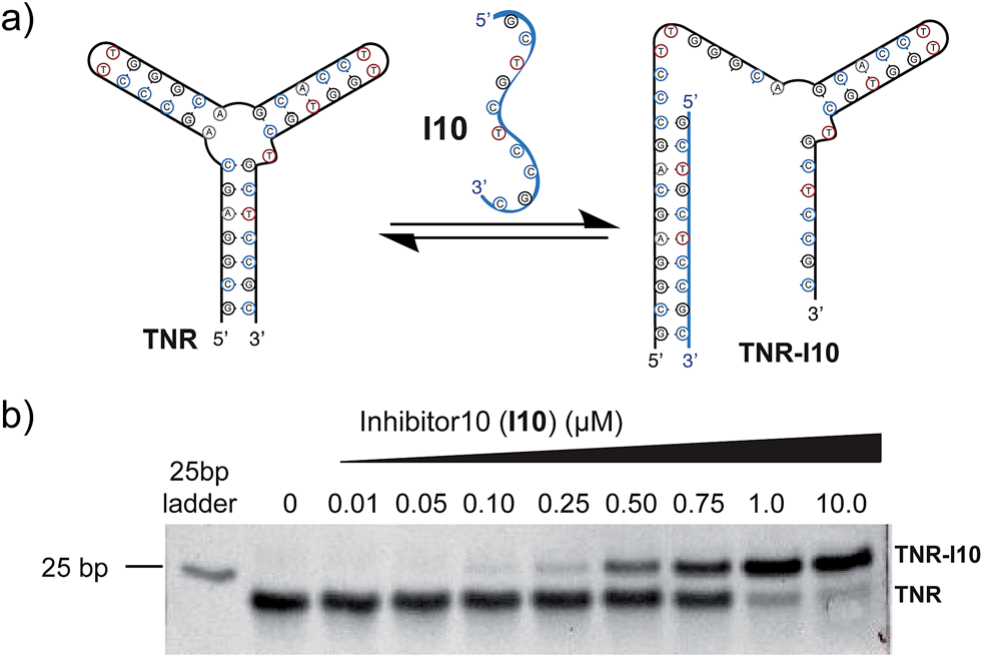
(a) Schematic of gel shift assay. (b) The folded **TNR 3WJ** was incubated with different concentrations of an inhibitor strand (**I10**) complementary to the 5′-end, resulting in formation of **TNR–I10**. Non-denaturing polyacrylamide gel ran in 1× TBE buffer at 4 °C.

**Fig. 3:**
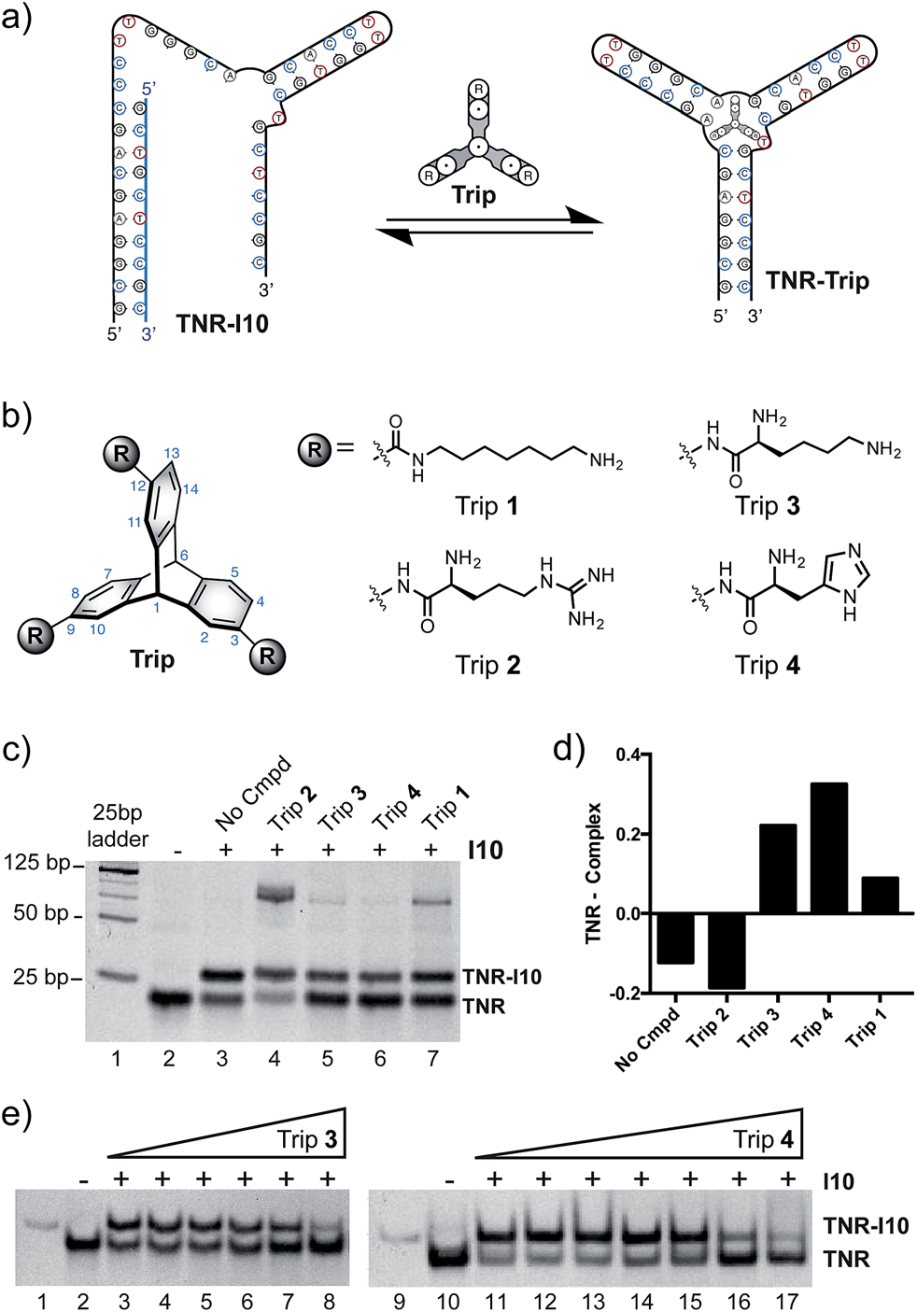
(a) Schematic of gel shift assay. The folded **TNR 3WJ** was incubated with an inhibitor strand complementary to the 5′-end, opening the junction structure (**TNR–I10**). Addition of triptycene results in reformation of the junction (**TNR–Trip**). (b) Structures of triptycene derivatives (Trip **1–4**). (c) Gel shift assay where **TNR–I10** was incubated with triptycene derivatives at a constant concentration. (d) A plot of the difference in band intensities of **TNR** and **TNR–I10**. Bars below zero in the plot indicated an increased amount of complex relative to 3WJ. (e) Gel shift assay in the presence and absence of Trip **3** and Trip **4**. Samples contained 0.5 μM **TNR** alone (where minus sign is indicated) or 0.5 μM **TNR** and 1.5 μM **I10** (where a plus sign is indicated). Increasing concentrations of Trip **3** were added (lane 3, 0 μM; lane 4, 0.01 μM; lane 5, 0.10 μM; lane 6, 0.50 μM; lane 7, 1.0 μM; lane 8, 5.0 μM) and Trip **4** (lane 11, 0 μM; lane 12, 0.01 μM; lane 13, 0.10 μM; lane 14, 0.50 μM; lane 15, 1.0 μM; lane 16, 5.0 μM; lane 17, 10.0 μM). Lanes 1 and 9 are loaded with a 25 base pair DNA ladder in which the band present corresponds to 25 bases. Free **TNR** junction and **TNR–I10** complex are indicated. Non-denaturing polyacrylamide gel ran in 1× TBE buffer at 4 °C.

Utilizing a well-studied (CAG)·(CTG) repeat sequence known to form slipped-DNA three-way junctions, we developed a competitive inhibitor-based gel shift assay. This assay was inspired by previous work from our laboratory and seminal studies from the Plaxco and Ricci laboratories.^1i,7^ We utilized the junction forming (CAG)·(CTG) repeat sequence (**TNR**) and an inhibitor strand (**I10**) shown in Fig. 2a. The optimal inhibitor strand was 10 base pairs long and complementary to the 5′-end of the junction. Titration of inhibitor strand **I10** into the folded junction resulted in a concentration dependent supershift of the band corresponding to hybridization of the **TNR** junction with **I10**, forming a larger molecular weight complex (**TNR–I10**) (Fig. 2b). Addition of small molecules capable of binding the **TNR** junction leads to **I10** strand displacement from the **TNR–I10** complex in a concentration dependent manner (Fig. 3a).

Inspired by nucleic acid binding proteins, we synthesized a new class of triptycene molecules containing amino acids commonly found at the protein–nucleic acid interface. Analysis of these interfaces reveals an abundance of positively charged amino acid residues such as arginine, lysine, and histidine. We synthesized triptycene derivatives substituted with arginine, lysine, and histidine (Trip **2–4**) as shown in Fig. 3b. Compounds **2–4** were assessed using a competitive displacement gel shift assay and compared to Trip **1**, which was previously shown to significantly stabilize 3WJs (Fig. 3). Folded **TNR 3WJ** was incubated with **I10**, followed by addition of triptycenes **1–4** at the same concentration. Triptycenes **1–4** were able to reform the junction to varying degrees (Fig. 3c and d). Trip **3** and Trip **4** were the most effective, while Trip **1** was slightly less effective and Trip **2** did not show reformation of the junction. Interestingly, addition of Trip **1** and Trip **2** resulted in a slower moving band on the gel, indicating formation of a higher order structure. Trip **3** and Trip **4** resulted in the most significant inhibitor (**I10**) displacement, shifting **TNR–I10** to the **TNR–Trip** complex and reforming the junction. A full titration of Trip **3** and Trip **4** with pre-incubated **TNR–I10** was then performed (Fig. 3e). Concentration dependent displacement of the inhibitor strand (**I10**) resulted in reformation of the junction.

**Fig. 4:**
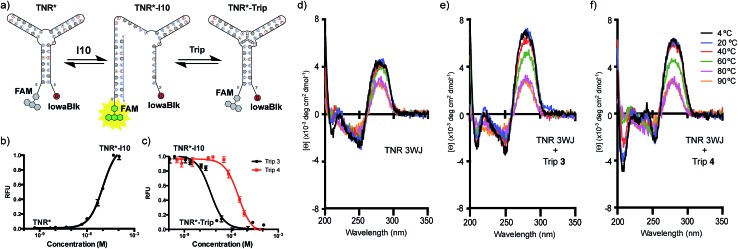
Fluorescence-quenching assay and circular dichroism (CD). (a) **TNR 3WJ** labeled with a fluorophore and quencher. When folded, low fluorescence is observed. Addition of inhibitor **I10** opens the junction, resulting in an increase in fluorescence (TNR*–I10). Addition of triptycene reforms the junction, resulting in quenching of fluorescence. (b) Titration of **I10** to the folded junction results in an increase in fluorescence. Fluorescence assay was conducted in 50 mM sodium phosphate buffer at pH 7.2. (c) Titration of Trip **3** and Trip **4** to TNR*–I10 results in a decrease in fluorescence. (d) Temperature-dependent circular dichroism of the **TNR** junction. Temperature-dependent CD in the presence of Trip **3** (e) and Trip **4** (f). Circular dichroism measurements were conducted in 50 mM sodium phosphate buffer at pH 7.2.

Next, we tested the ability of Trip **3** and **4** to induce fluorescence-quenching upon junction formation using a double labelled (CAG)·(CTG) repeat oligonucleotide. The **TNR** oligonucleotide was labelled with a FAM fluorophore on the 5′-end and an IowaBlack quencher on the 3′-end (TNR*) (Fig. 4a). Formation of the junction results in little to no fluorescence due to the proximity of the quencher and fluorophore, resulting in efficient contact quenching. The junction was incubated with the 10 bp inhibitor strand (**I10**). As expected, titration of **I10** resulted in an increase in fluorescence, consistent with disruption of the folded junction, in which the fluorophore and quencher are separated in space (TNR*–I10) (Fig. 4b). Pre-formation of the open inhibited state of the junction (TNR*–I10) followed by titration of Trip **3** or Trip **4**, resulted in a concentration dependent decrease in fluorescence (Fig. 4c). The decrease in signal indicates that the fluorophore and quencher are in close proximity due to refolding of the junction. Due to the competitive nature of this assay, only apparent *K*_d_ values are reported. The apparent *K*_d_ values of Trip **3** and Trip **4** were determined to be 52.8 nM and 2.36 μM, respectively.

Temperature-dependent circular dichroism (CD) was used to further characterize the interaction of Trip **3** and Trip **4** with the **TNR** junction. The CD spectra are indicative of B-DNA, showing positive signals at 280 nm due to base stacking and negative signals at 250 nm due to the right-handed helicity (Fig. 4d–f).^8^ As the temperature increased, the positive band at 280 nm decreased and the negative band at 250 nm increased, consistent with melting of the DNA structure (Fig. 4d). Upon addition of Trip **3** and Trip **4**, distinct spectral changes are observed, resulting in an increased signal at 240 nm as well as a more positive signal at 280 nm and more negative signal at 210 nm (Fig. 4e and f). The increase at 280 nm is consistent with enhanced base stacking and increased helicity. Studies have shown that CAG slip-outs in a 3WJ are less paired and adopt more of an open loop structure.^9^ The increased helicity observed in the CD spectrum may be due to increased base pairing interactions in the slip-out region upon addition of Trip **3** and Trip **4**.

## Conclusions

In summary, we have described triptycene-based molecules that bind to d(CAG)·(CTG) trinucleotide repeats. Trip **3** and Trip **4** bind to a model (CAG)·(CTG) repeat as determined by gel shift and fluorescence-quenching experiments. The CD spectra are also consistent with enhanced helicity of the slipped out junction upon addition of Trip **3** and **4**. This new class of nucleic acid binding small molecule may serve as inspiration for creating valuable probes for diseases associated with trinucleotide repeat expansions. Trinucleotide repeat nucleic acid sequences are associated with a large number (>30) of inherited human muscular and neurological diseases. The trinucleotide repeat tract length is dynamic and often correlates with disease severity, where short stable tracts are commonplace in the non-affected population. Longer unstable triplet repeat tracts are more prone to expansion as opposed to contraction, in addition to being predisposed to generational transmission.^6*c*^ Trinucleotide repeat repair outcomes are also affected by structural features present in slipped sequences, where the structure may determine which proteins are recruited for repair.^10^ Stabilization of a particular structure could lead to altered repair of slipped-out junctions. Addition of ligands that bind to these junctions may affect repair outcomes as well as recruitment of proteins. Small molecule probes will provide valuable tools to study these processes. Small molecule binding and stabilization or modulation of these dynamic structures could lead to the development of therapeutic agents for their associated diseases.
