# Supplementary material for: Triptycene-based small molecules modulate (CAG)·(CTG) repeat junctions
Source: Chem Sci. 2015 Jun 10;6(8):4752–5. doi: 10.1039/c5sc01595b (PMC4538686; doi:10.1039/c5sc01595b)
Supplement: Supplementary file 1 [file SC-006-C5SC01595B-s001.pdf]

Electronic Supplementary Material (ESI) for Chemical Science.
This journal is © The Royal Society of Chemistry 2015

Triptycene-Based Small Molecules Modulate (CAG)·(CTG) Repeat Junctions

Stephanie A. Barros and David M. Chenoweth*

Department of Chemistry, University of Pennsylvania, 231 South 34th Street, Philadelphia, Pennsylvania 19104-6323, United States

Correspondence and requests for materials should be addressed to D.M.C. (email: dcheno@sas.upenn.edu).

Table of Contents

General Methods……………………………………………………………………….....S2

Synthesis……………………………………………………………………………….S2-S4

HPLC Analysis……….………………..……………………………………..…………..S4-S5

NMR Spectra………………………………………………………………………..……S6-S9

Gel Shift Experiments………………………………………………………….…...S10

Fluorescence Quenching Experiments…………………………………………….....…S10

Circular Dichroism………………………………………………………………….S10

General Methods:

TNR DNA 3WJ (5'-GCGGAGCAGCCCTTGGGCAGCACCTTGGTGCTGCTCCGC-3') and DNA inhibitor 10 (5'-GCTGCTCCGC-3') were purchased from Integrated DNA Technologies (IDT). HPLC-purified TNR DNA 3WJ oligo modified with a 5'-FAM and a 3'-IowaBlack was purchased from IDT. Amino acids (Boc-Arg(Mtr)-OH, Boc-Lys(Boc)-OH, and Boc-His-OH were purchased from Merck Millipore Novabiochem (Billerica, MA, USA). (1-[Bis(dimethylamino)methylene]-1H-1,2,3-triazolo[4,5-b]pyridinium 3-oxid hexafluorophosphate) (HATU) was purchased from GenScript (Piscataway, NJ, USA). All other reagents were purchased from Sigma Aldrich (St. Louis, MO, USA) and used without further purification. Reactions requiring anhydrous conditions were run under argon with solvents purchased from Fisher dried via an alumina column. Thin-layer chromatography was done using Sorbent Technologies (Norcross, GA, USA) silica plates (250 μm thickness). Milli-Q (18 MΩ) water was used for all solutions (Millipore; Billerica, MA, USA).

^{1}H and ^{13}C NMR were recorded on a Bruker UNI 500 NMR at 500 and 125 MHz, respectively. Matrix-assisted laser desorption ionization (MALDI) mass spectra were obtained on a Bruker Ultraflex III MALDI-TOF-TOF mass spectrometer (Billerica, MA, USA) using α-cyano-4-hydroxycinnamic acid (CHCA). Low resolution electrospray ionization (ESI) mass spectra (LRMS) were collected on a Waters Acquity Ultra Performance LC (Milford, MA, USA). High resolution mass spectra were obtained at the University of Pennsylvania Mass Spectrometry Center on a Waters LC-TOF mass spectrometer (model LCT-XE Premier) using electrospray ionization in positive or negative mode, depending on the analyte. High-performance liquid chromatography was performed on a JASCO HPLC (Easton, MD, USA) equipped with a Phenomenx (Torrance, CA, USA) column (Luna 5μ C18(2) 100A; 250 x 4.60 mm, 5 μm) using aqueous (H2O + 0.1% CF3CO2H) and organic (CH3CN) phases. Circular dichroism experiments were performed on a JASCO J-1500 CD Spectrometer (Easton, MD, USA) using a 0.1 cm path length quartz cuvette. Fluorescence measurements were collected on a Tecan M1000 plate reader (Mannedorf, Switzerland). HPLC chromatograms were obtained at all wavelengths from 200 to 800 nm (bottom plot). 254 and 214 nm were chosen as virtual channels to show the absorbances at those two specific wavelengths (top plot). The blue line corresponds to 254 nm and the red line corresponds to 214 nm (top plot). The lamps used were D2 + W with a slit width of 4 nm. A flow rate of 1.00 mL/min was used over 35 minutes. The method began at 10 % acetonitrile and 90 % water + 0.1 % TFA. The gradient was increased to 25 % acetonitrile over 25 minutes and then increased to 100% acetonitrile.

Synthesis:

General Procedure:

To a solution of **5**[1] (0.08 mmol) in DMF (1 mL) was added HATU (0.256 mmol) and DIEA (0.496 mmol) and was stirred at room temperature for 5 minutes. The corresponding amino acid (0.256 mmol) was added and stirred overnight. The reaction mixture was concentrated under vacuum, then diluted with water and extracted with EtOAc. The crude

product was suspended in 4M HCl in dioxane and stirred for 2 hours. The mixture was concentrated, dissolved in acidic water (0.1% TFA) and washed with CH_2Cl_2. The product was purified on a JASCO High-Performance Liquid Chromatography (HPLC) instrument on a C18 column using aqueous (H_2O + 0;1% TFA) and organic (CH_3CN) phases.

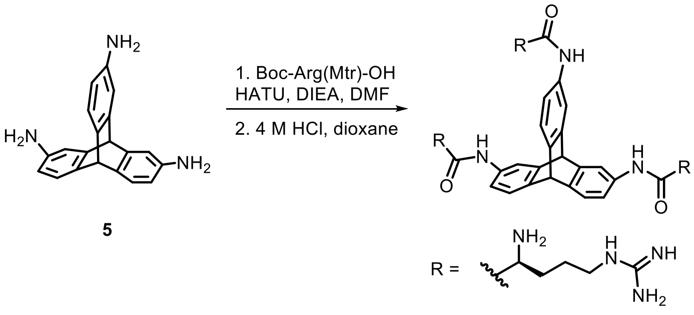

5

Trip **2**

(2S,2'S,2''S)-*N*,*N'*,*N''*-(9,10-dihydro-9,10-[1,2]benzenoanthracene-2,7,15-triyl)tris(2-amino-5-guanidinopentanamide) (2). ^{1}H NMR (500 MHz, D_2O) δ 7.65 (s, 3 H), 7.51-7.50 (d, 3 H) 7.12-7.10 (d, 3 H), 5.72 (s, 1 H), 5.66 (s, 1 H), 4.13-4.10 (t, 3 H), 3.23-3.20 (t, 3 H), 2.03-1.98 (m, 6 H), 1.72-1.66 (m, 6 H); HRMS *m/*z calcd for $C_{38}H_{56}N_{15}O_3^{3+}$ $[M+3H]^{3+}$ 256.8225, observed 256.8231.

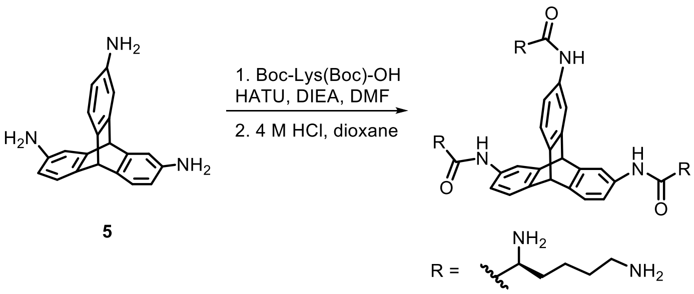

5

Trip **3**

(2S,2'S,2''S)-*N*,*N'*,*N''*-(9,10-dihydro-9,10-[1,2]benzenoanthracene-2,7,15-triyl)tris(2,6-diaminohexanamide) (3). ^{1}H NMR (500 MHz, D_2O) δ 7.65 (s, 3 H), 7.52-7.50 (d, 3 H) 7.13-7.11 (d, 3 H), 5.73 (s, 1 H), 5.67 (s, 1 H), 4.11-4.09 (t, 3 H), 2.99-2.96 (t, 6 H), 2.01-1.96 (m, 6 H), 1.17-1.67 (m, 6 H), 1.51 (m, 6H); HRMS *m/z* calcd for $C_{38}H_{54}N_9O_3^{+}$ $[M+H]^{+}$ 684.4344, observed 684.4357.

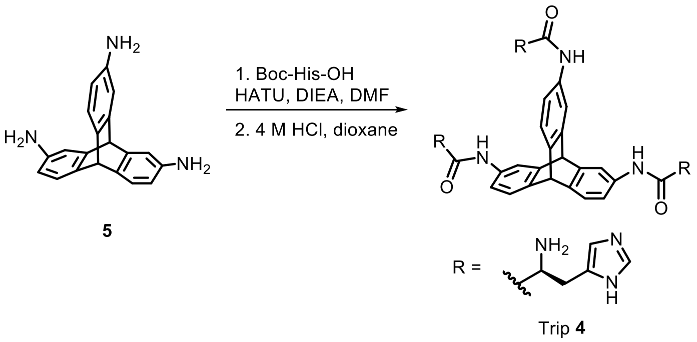

(2S,2'S,2''S)-*N*,*N'*,*N''*-(9,10-dihydro-9,10-[1,2]benzenoanthracene-2,7,15-triyl)tris(2-amino-3-(1H-imidazol-5-yl)propanamide) (4). ^{1}H NMR (500 MHz, D_2O) δ 8.58 (s, 3 H), 7.65 (s, 3 H) 7.54-7.52 (d, 3 H), 7.40 (s, 3 H), 7.07-7.05 (d, 3 H), 5.75 (s, 1 H), 5.69 (s, 1 H), 4.40-4.36 (t, 3 H), 3.47-3.45 (d, 6 H); ^{13}C NMR (500 MHz, D_2O) δ 166.63, 145.73, 142.70, 134.65, 133.19, 126.72, 124.29, 118.65, 118.15, 117.52, 115.20, 52.93, 51.60, 26.58; MALDI-TOF *m/z* calcd for $C_{38}H_{38}N_{12}O_3$ [M+H] 710.80, observed 711.397, HRMS *m/z* calcd for $C_{38}H_{39}N_{12}O_3^+$ $[M+H]^+$ 711.3263, observed 711.3274.

HPLC:

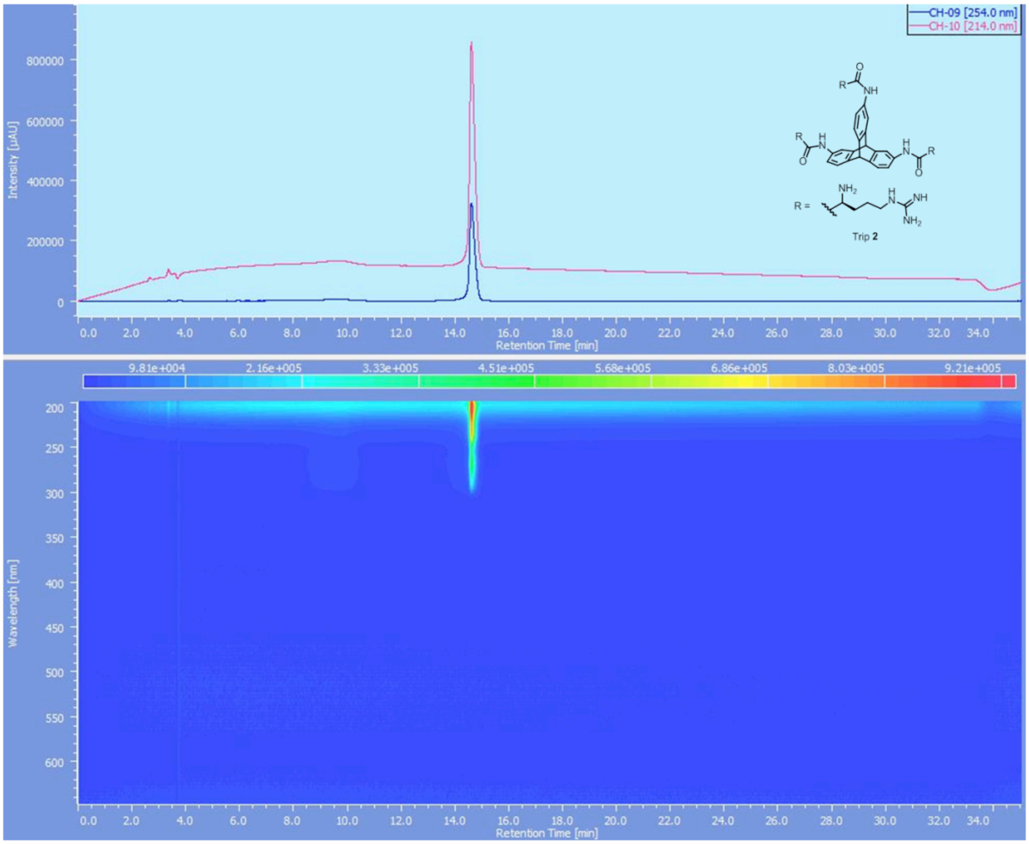

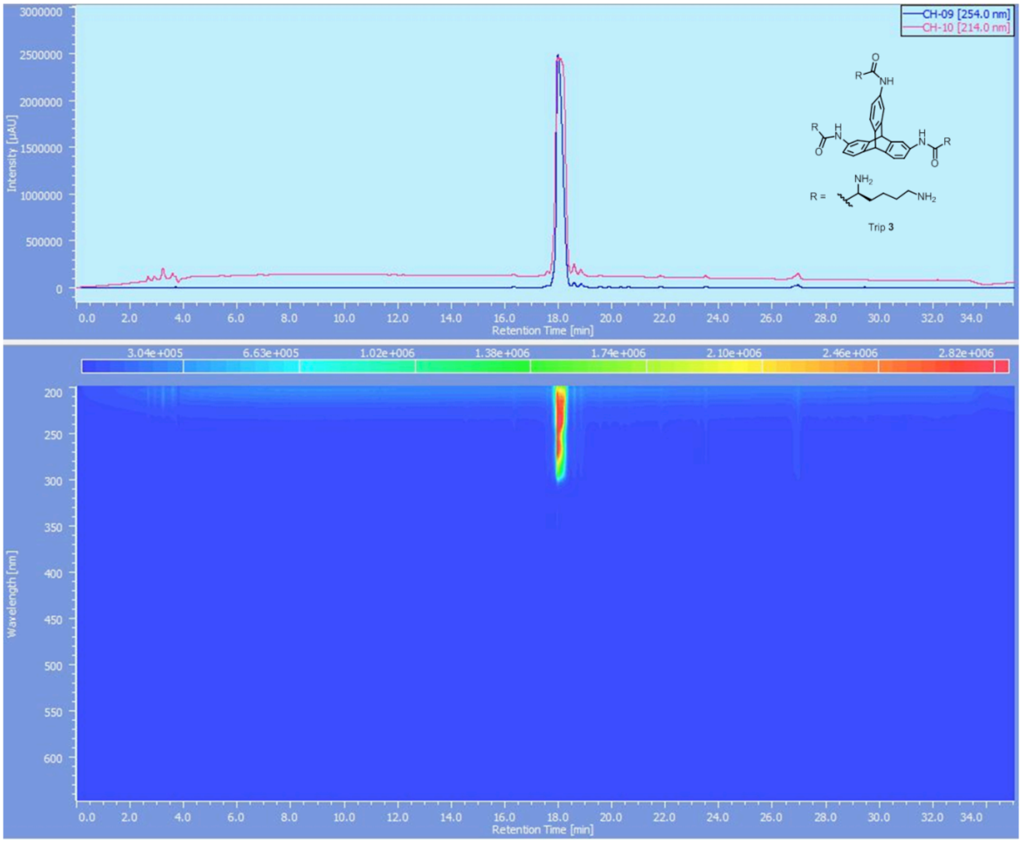
CH-09 [254.0 nm]
CH-10 [214.0 nm]
Intensity [µAU]
Retention Time [min]
Wavelength [nm]
R =
Trip 3

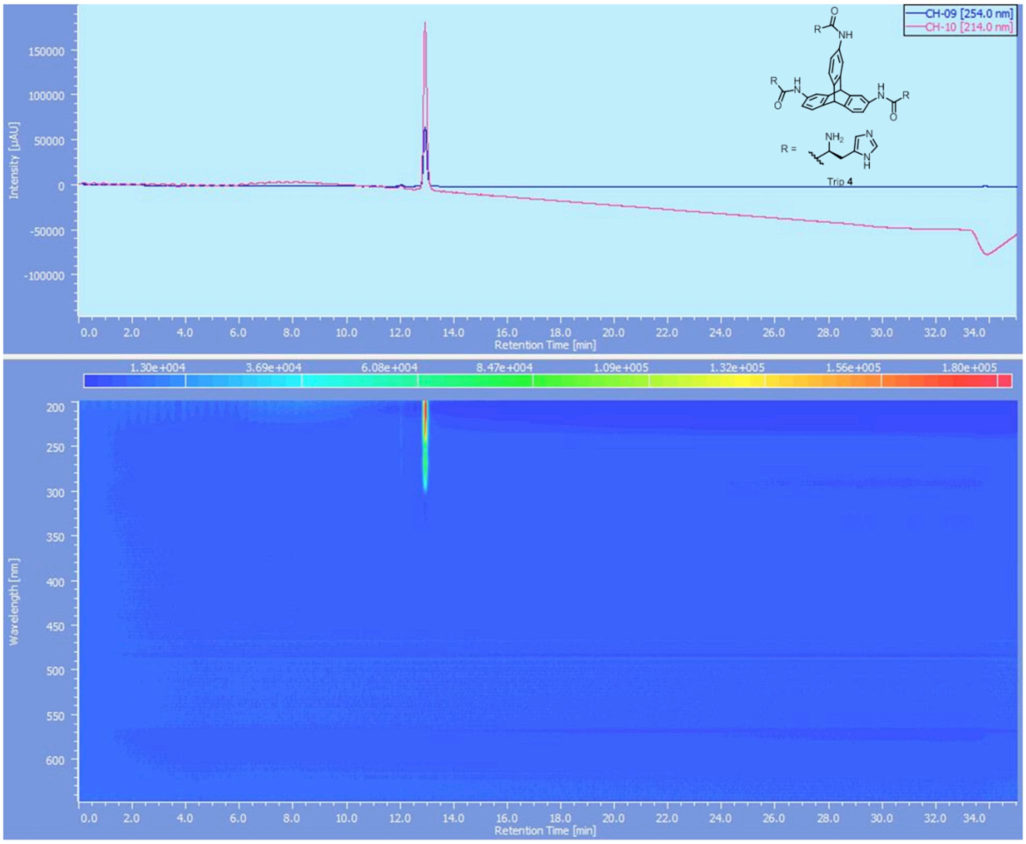
CH-09 [254.0 nm]
CH-10 [214.0 nm]
Intensity [µAU]
Retention Time [min]
Wavelength [nm]
R =
Trip 4

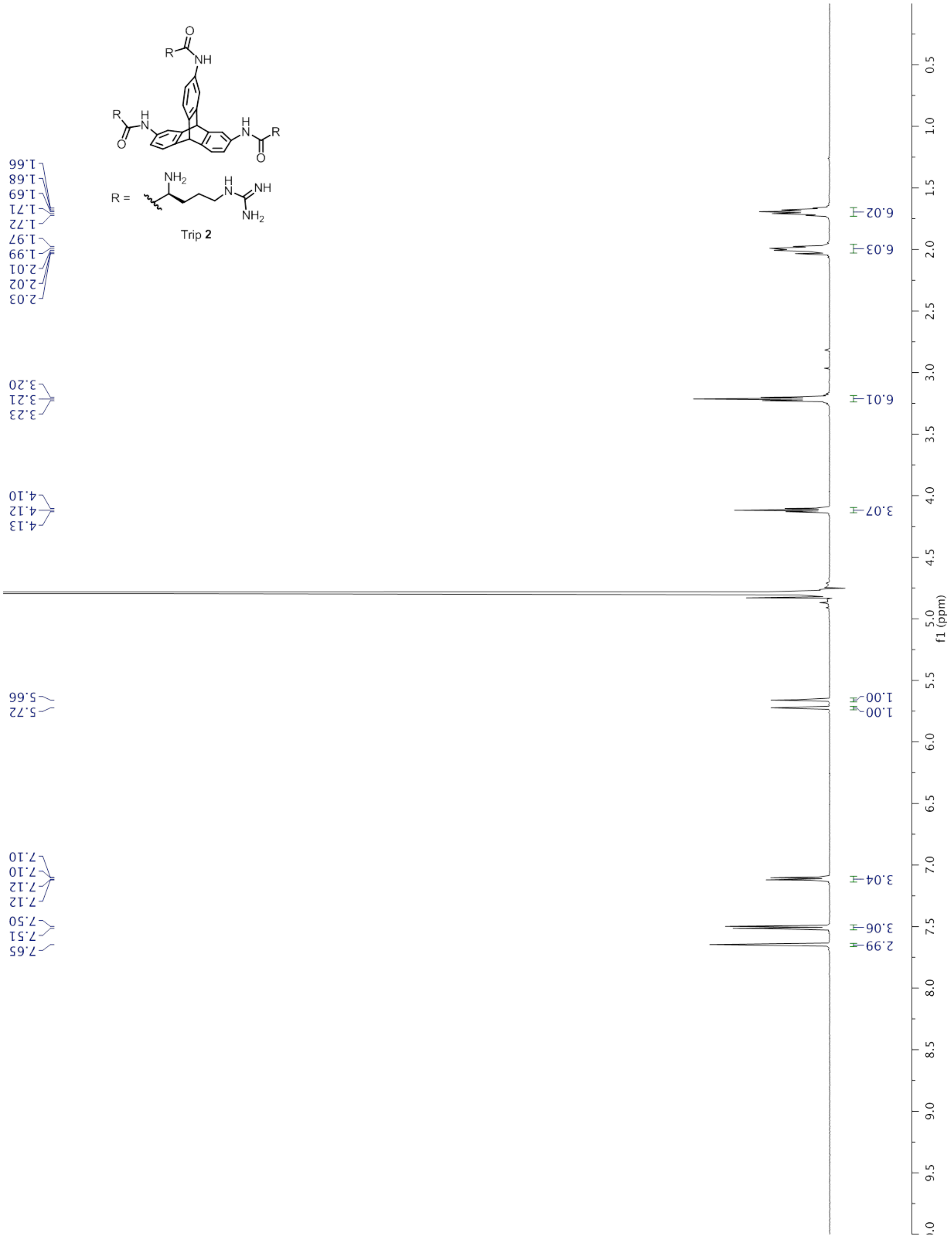
O
R
NH
H
N
R
O
NH2
H
N
NH
NH2
R =
Trip 2
1.66
1.68
1.69
1.71
1.72
1.97
1.99
2.01
2.02
2.03
3.20
3.21
3.23
4.10
4.12
4.13
5.66
5.72
7.10
7.10
7.12
7.12
7.50
7.51
7.65
6.02
6.03
6.01
3.07
1.00
1.00
3.04
3.06
2.99
f1 (ppm)

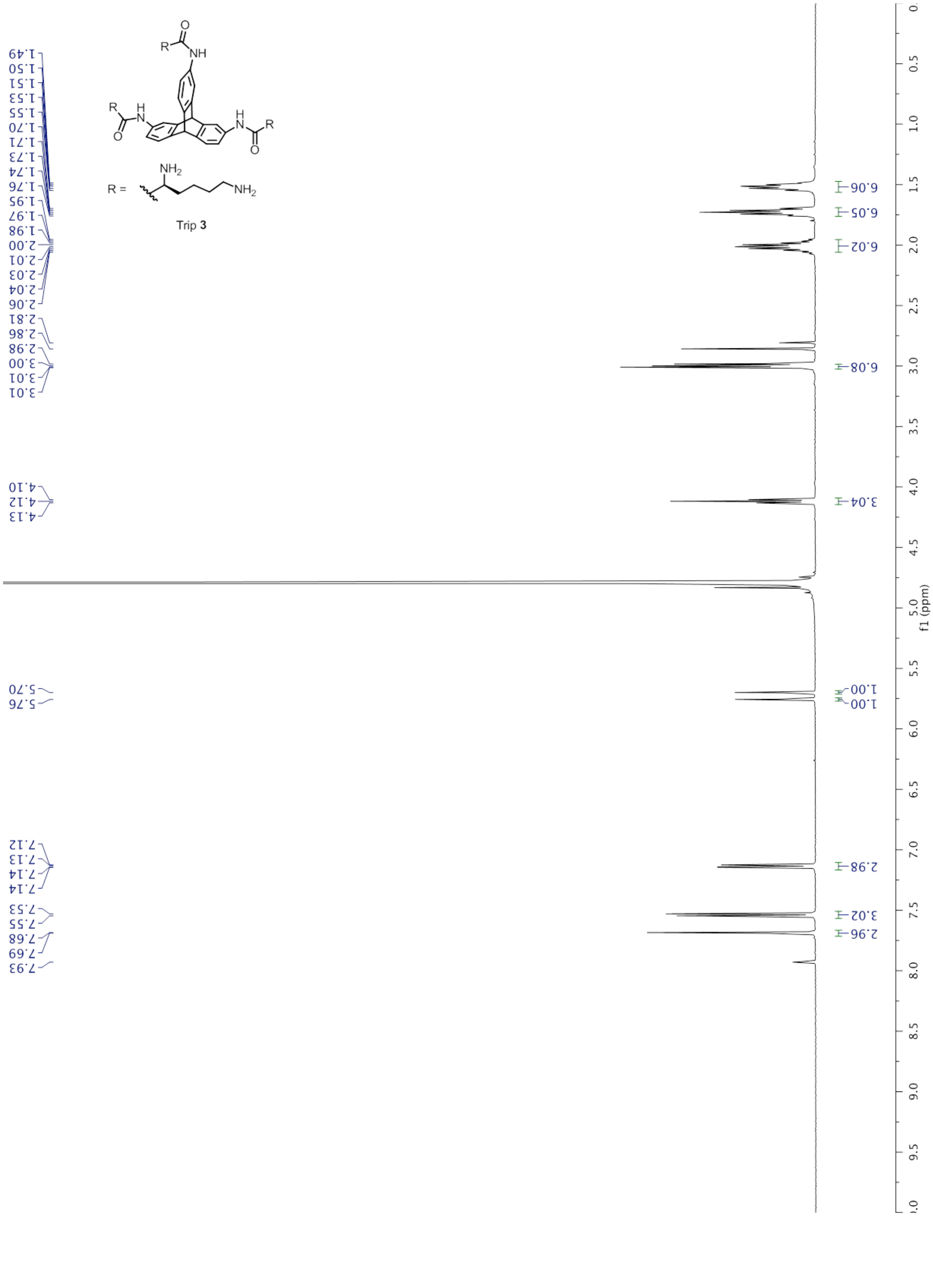
R =
NH2
NH2
Trip 3
f1 (ppm)
1.49 1.50 1.51 1.53 1.55 1.70 1.71 1.73 1.74 1.76 1.95 1.97 1.98 2.00 2.01 2.03 2.04 2.06 2.81 2.86 2.98 3.00 3.01 3.01
4.10 4.12 4.13
5.70 5.76
7.12 7.13 7.14 7.14 7.53 7.55 7.68 7.69 7.93
6.06 6.05 6.02 6.08 3.04 1.00 1.00 2.98 3.02 2.96

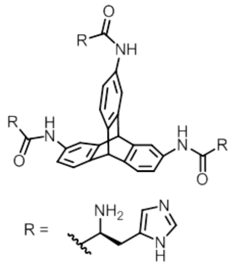

R
NH
H
N
O
R =
NH2
N
H

Trip **4**

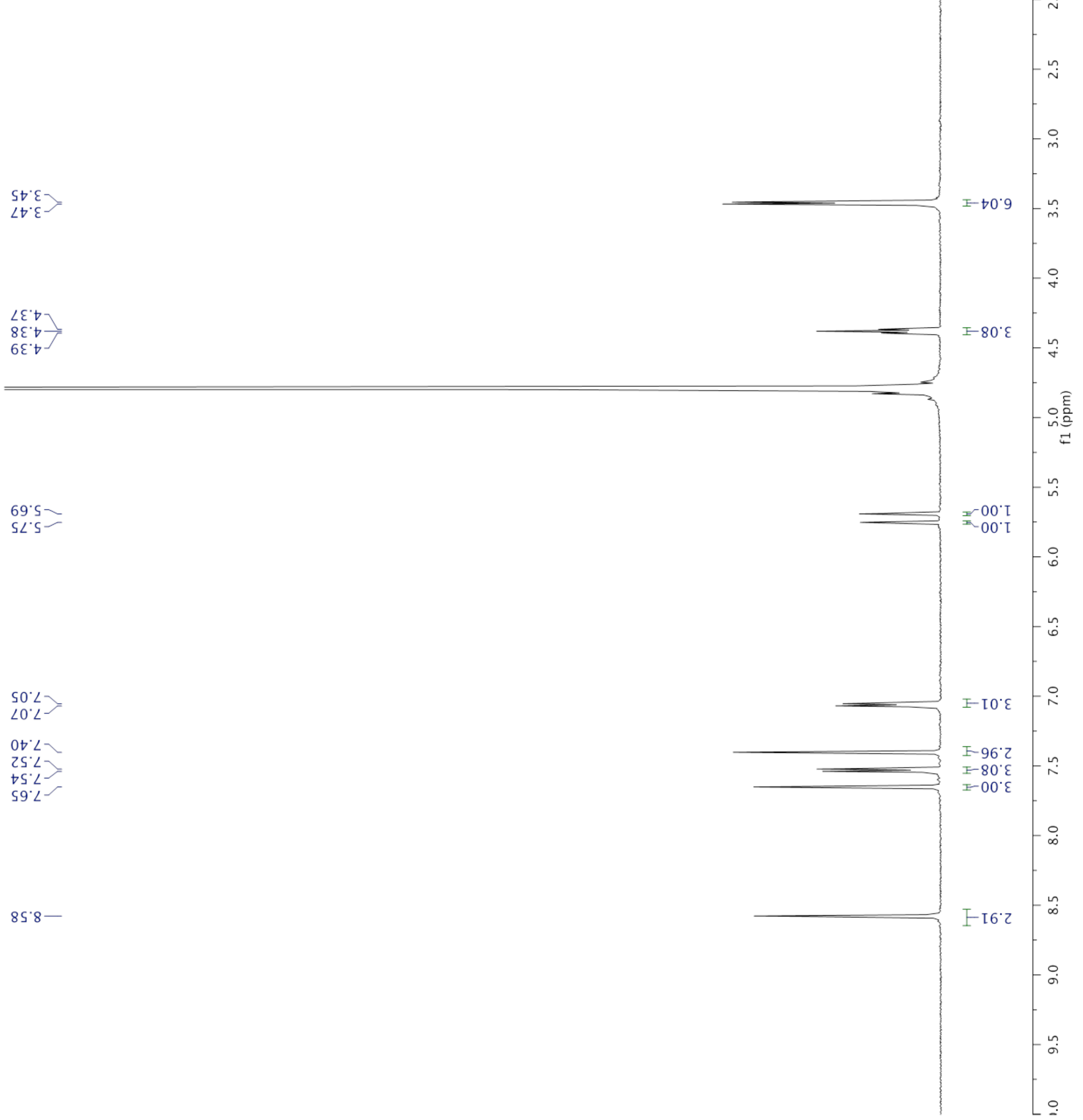

3.45
3.47
4.37
4.38
4.39
5.69
5.75
7.05
7.07
7.40
7.52
7.54
7.65
8.58
6.04
3.08
1.00
1.00
3.01
2.96
3.08
3.00
2.91
f1 (ppm)

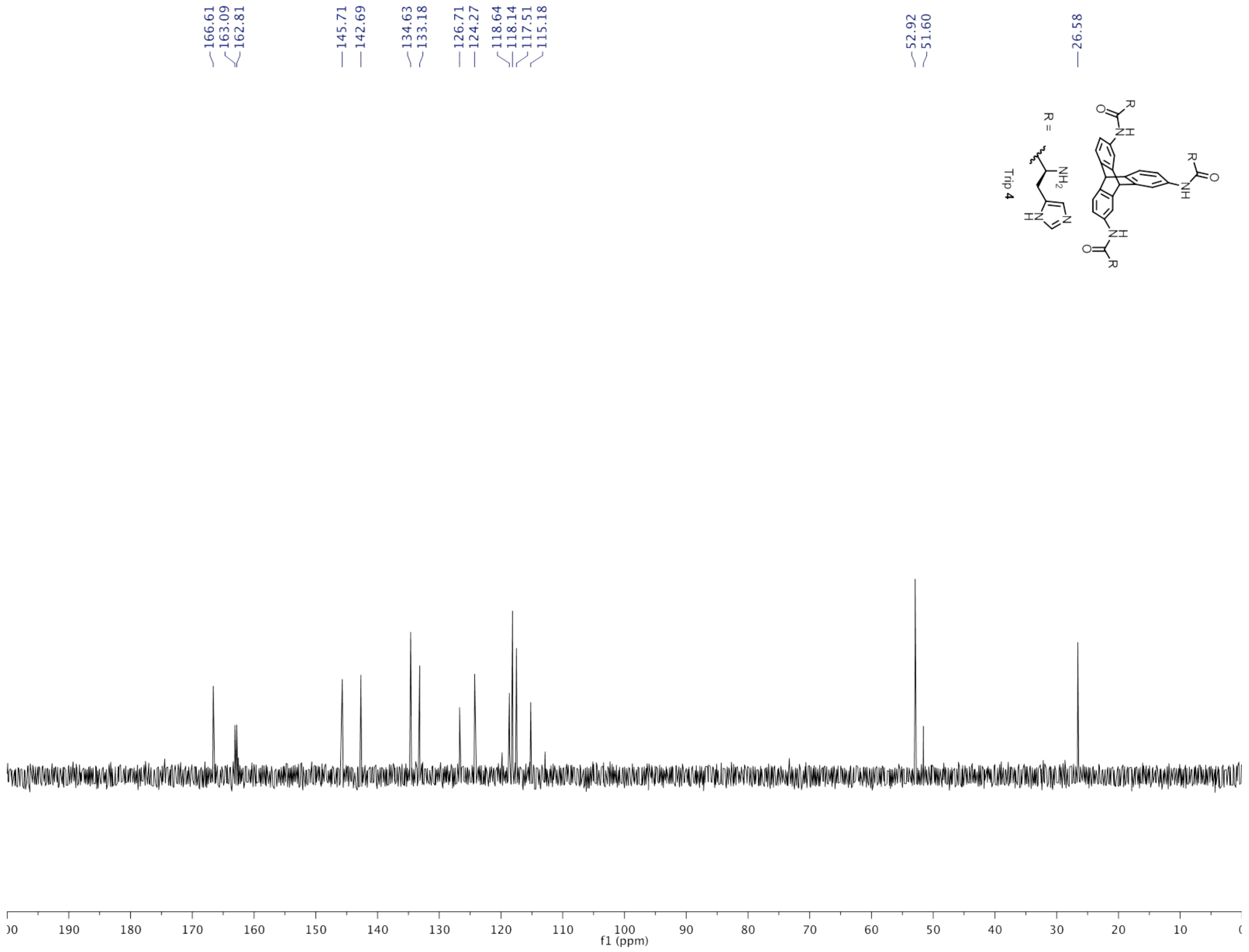
166.61
163.09
162.81
145.71
142.69
134.63
133.18
126.71
124.27
118.64
118.14
117.51
115.18
52.92
51.60
26.58
R =
NH2
Trip 4
f1 (ppm)

Gel Shift Assay:

All gel shift experiments were conducted in 50 mM sodium phosphate buffer, pH 7.2. The screening gel was run by incubating TNR 3WJ (0.5 μM) with inhibitor strand 10 bases long (1.5 μM) in a 20 μL solution at room temperature for 2 hours. Triptycenes were then added at a final concentration of 10 μM and incubated for 2 hours. Samples were run on a 20% non-denaturing polyacrylamide gel (19:1 monomer:bis) at 50V in 1X TBE buffer at 4 °C for 10 hours. Gels were stained with SYBR Gold for 10 minutes and visualized using a BioRad GelDoc XR+ imager.

Inhibitor strand titration gel was run by incubating TNR 3WJ (0.5 μM) with increasing concentrations of inhibitor strand in a 20 μL solution at room temperature for 2 hours. Samples for compound titration were prepared by incubating TNR 3WJ (0.5 μM) with inhibitor strand (1.5 μM) for 2 hours followed by titration of Trip **4** and incubation at room temperature for 2 hours. Samples were run on a gel as described above.

Fluorescence quenching experiments:

All binding experiments were conducted in 50 mM sodium phosphate buffer, pH 7.2. Fluorescence measurements were recorded with excitation at 495 nm and emission at 520 nm using 5 nm bandwidths. Inhibitor strand binding curves were obtained by adding 1 μL of increasing concentrations of inhibitor strand to 19 μL of 120 nM TNR 3WJ. Samples were incubated for 2 hours and ran in triplicate in a 384-well plate. Inhibitor strand displacement curves were obtained by incubating 120 nM aptamer with 1 μM inhibitor 10 for 2 hours, followed by addition of increasing concentrations of **4**. Samples were incubated for 2 hours and measured in triplicate.

Circular Dichroism:

DNA was suspended at 6 μM in 50 mM sodium phosphate buffer, pH 7.2 and annealed by heating to 90°C for 5 min, cooled to room temperature slowly, then to 4 °C. Spectra were measured every 0.5 nm between 350 nm and 200 nm with an 8 s averaging time. Samples were incubated at each temperature for 20 minutes prior to scan. Samples were incubated with ligand (24 μM) at room temperature for 1 hour. All CD spectra were buffer corrected and converted to molar ellipticity.

References

(1) S. A. Barros, D. M. Chenoweth, *Angew. Chem. Int. Ed.* **2014**, *53*, 13746-13750.
